# Achieved low-density lipoprotein cholesterol to high-density lipoprotein cholesterol ratio predicts the pathophysiological evolution of lipid-rich plaques in acute coronary syndromes: an optical coherence tomography study

**DOI:** 10.3389/fcvm.2023.1181074

**Published:** 2023-07-10

**Authors:** Luping He, Boling Yi, Dirui Zhang, Sining Hu, Chen Zhao, Rui Sun, Jianlin Ma, Jingbo Hou, Haibo Jia, Lijia Ma, Bo Yu

**Affiliations:** ^1^ Department of Cardiology, The 2nd Affiliated Hospital of Harbin Medical University, Harbin, China; ^2^ Key Laboratory of Myocardial Ischemia, Ministry of Education, Harbin Medical University, Harbin, China

**Keywords:** acute coronary syndrome, optical coherence tomography, lipid-rich plaques, plaque evolution, achieved serum lipid

## Abstract

**Background:**

As a novel lipoprotein ratio, baseline low-density lipoprotein cholesterol to high-density lipoprotein cholesterol ratio (LHR) is closely related to the clinical outcomes of acute coronary syndromes (ACS) after percutaneous coronary intervention. However, the pathophysiological impact of achieved LHR (aLHR) on the evolution of non-culprit lipid-rich plaques has not been systematically explored.

**Methods:**

Between September 2013 and December 2018, ACS patients with both baseline and 1-year follow-up optical coherence tomography (OCT) examinations were included in current study. They were divided into two groups according to the median value of aLHR at 1 year.

**Results:**

Overall, 132 patients with 215 lipid-rich plaques were enrolled, with a median aLHR: 1.62. There were thinner fibrous cap thickness (FCT) (133.3 [70.0–180.0] µm vs. 160.0 [100.0–208.3] µm, *p* = 0.025) and higher prevalence of thin-cap fibroatheroma (TCFA) (24 [22.4%] vs. 13 [12.0%], *p* = 0.044) and CLIMA-defined high-risk plaques (12 [11.2%] vs. 3[2.8%], *p* = 0.015) in the high aLHR group at 1 year. Compared with other serum lipid indexes, aLHR showed the best robust correlation with the evolution of plaque vulnerability in both unadjusted and adjusted analyses. Cut-off value of aLHR to predict the progression of maximal lipid arc and FCT was 1.51. In the adjusted model, aLHR ≥1.51 was an independent predictor of TCFA [odds ratio (OR): 3.008, 95% CI: 1.370 to 6.605, *p* = 0.006] at 1 year.

**Conclusions:**

aLHR correlates well with the evolution of lipid-rich plaques and vulnerable phenotypes at 1-year follow-up, which might be an important and convenient serum indicator in the secondary prevention of ACS.

## Introduction

Dyslipidemia plays an important role in the occurrence, development and secondary prevention of acute coronary syndromes (ACS) (1). Many studies have shown that not only the index blood lipid level, but also their variabilities and controlling levels after discharge are of great significance to the secondary prevention and long-term prognosis of cardiovascular disease (2–5). In addition to low-density lipoprotein cholesterol (LDL-C), high-density lipoprotein cholesterol (HDL-C) and triglyceride, several novel lipoprotein ratios have been proved with greater predictive power in cardiovascular prevention, especially the LDL-C to HDL-C ratio (LHR), which could reflect not only the atherogenic lipoprotein, but also the protective lipoprotein and their balance (6–9). Jesús Millán et al. pointed out that LHR was more predictive of cardiovascular disease risk than either lipoprotein alone (9). More recently, LHR was demonstrated to be a predictor of long-term prognosis clinical prognosis for ACS or ST-segment elevation myocardial infarction (STEMI) patients (10, 11). Besides, a recent study demonstrated a close association between achieved levels of atherogenic lipoproteins and the progression in percent atheroma volume through serial intravascular ultrasound examinations (2). However, the detailed impact of achieved LHR (aLHR) during follow-up on the evolution of lipid-rich plaques remains unknown.

Due to the high resolution and specific recognition of lipid components in-vivo, optical coherence tomography (OCT) has been widely used in the process of coronary interventions. It can not only quantitatively assess luminal features of targeted lesions, but also the lipid arc and fibrous cap thickness (FCT), which are vital features of plaque vulnerability and closely related to adverse clinical outcomes (12–14). The purpose of this study was to explore the relationship between aLHR and the progression of lipid-rich plaques in ACS at 1-year follow-up.

## Methods

Patients who underwent baseline and follow-up coronary OCT examinations at the Second Affiliated Hospital of Harbin Medical University September 2013 to December 2018 were retrospectively enrolled in current study. Decisions to perform OCT examinations and detailed scanning vessels were decided by the operators during percutaneous coronary intervention (PCI). The main inclusion criteria were as follows: (1) patients diagnosed as ACS at baseline, including STEMI and non-ST-segment elevation acute coronary syndrome (15); (2) Time duration between baseline and follow-up was 12 ± 3 months, to minimize the interference of time on plaque progression; (3) de-novo non-culprit lipid-rich plaques. Major exclusion criteria were: (1) patients with incomplete data records of LDL-C or HDL-C at baseline or follow-up; (2) suboptimal OCT images quality of the interested region. This study followed the Helsinki Declaration and was approved by the ethics committee of the Second Affiliated Hospital of Harbin Medical University.

### Clinical and serum lipid data collection

Baseline and 1-year follow-up clinical data, including demographic characteristics, cardiovascular risk factors, results of laboratory specimens, medications were obtained from the electronic medical record system of our hospital. The achieved lipid levels were collected and tested from the fasting venous blood samples during hospitalization of 1-year follow-up.

### OCT analysis

OCT images were analyzed by experienced investigators at the intravascular imaging core laboratory of the Second Affiliated Hospital of Harbin Medical University. Culprit lesions were identified by the comprehensive evaluation of OCT images, angiographic findings, electrocardiograms and echocardiography results. Non-culprit lesions were identified by OCT as a loss of the normal three-layered structure of the coronary wall in at least 3 consecutive 1-mm cross-sections (16). A distance of over 5 mm was recognized as different plaques. Typical landmarks, such as bifurcations, calcifications and micro-structures was used to ensure reliable matches and comparisons baseline and follow-up OCT images. Lipid pool was characterized as a signal-poor region with diffuse border under the signal-rich fibrous cap. Lipid-rich plaques were defined as plaques with a maximum lipid arc over 90°. Quantitative analysis of the lumen area and lipid pool was conducted at 1-mm intervals on cross-sectional OCT images. Length of the lipid pool was measured on the longitudinal view. FCT was measured 3 times at its thinnest part and the mean value was calculated. According to the FCT, lipid-rich plaques were further divided as thin-cap fibroatheroma (TCFA) with FCT ≤ 65 µm and thick-cap fibroatheroma with FCT > 65 µm. According to the CLIMA study, high-risk plaque was defined as the simultaneous presence of minimal lumen area (MLA) < 3.5 mm^2^, FCT < 75 µm, maximum lipid arc >180° and OCT-defined macrophages (17). Distance to the ostium was measured from MLA of the non-culprit lesion to the ostium of the involved coronary. For detailed qualitative and quantitative analysis protocols, please refer to the published articles (18, 19). There were excellent intra-observer and inter-observer agreement (kappa value) for the identification of TCFA (0.853 and 0.856, respectively) and microchannel (0.948 and 0.894, respectively), and substantial agreement for macrophage (0.758 and 0.659, respectively). The changes (▵ value) in lumen area, lesion length, lipid arc, lipid length and FCT were calculated as their numerical values at follow-up minus the corresponding baseline values.

### Statistical analysis

The SPSS 26.0 (IBM Corp., Armonk, NY, USA) was used for statistical analysis. Continuous variables were tested for normality with the Kolmogorov–Smirnov test. Normally distributed variables were reported as mean ± standard deviation (SD) and compared with the Student's *t*-test. Non-normally distributed variables were expressed as medians (quartiles) and compared with Mann–Whitney *U*-test. Categorical variables have been shown as absolute frequencies (relative percentages) and compared with the Chi-square test or Fisher's exact test. Kappa test was conducted to assess the intra- and inter-observer agreement. Pearson correlation analysis and partial correlations analysis were used to examine the associations of serum lipid levels with OCT-detected plaque evolutions. The area under the curve (AUC) was compared using receiver operating characteristic (ROC) analysis to determine the best cut-off value of aLHR to be used for predicting lesion regression within 1 year. To account for the potential effects of clustering of multiple non-culprit lipid-rich plaques in a single patient, generalized estimating equations were used to identify independent predictors for vulnerable plaque phenotypes at 1 year. A two-sided *p* < 0.05 was considered statistically significant.

## Results

### Baseline characteristics

Between September 2013 and December 2018, a total of 132 ACS patients with 215 non-culprit lipid-rich plaques were taken into final analysis. According to the median value of the aLHR (median value: 1.62), patients were divided into the low aLHR group and the high aLHR group separately. The median follow-up time duration of overall subjects was 366.5 (from 285 to 443) days, which was comparable between the two groups (366.0 [360.0–375.3] vs. 367.5 [357.3–377.0], *p* = 0.920).

Baseline demographic characteristics, cardiovascular risk factors and medications at discharge was shown in Table 1. No significant differences between the two groups were observed in these characteristics, except for a higher prevalence of dyslipidemia in the high aLHR group (48 [72.7%] vs. 37 [56.1%], *p* = 0.046). Five patients didn't have pre-dilation OCT images of the culprit vessel. Culprit lesion etiology was comparable between the two groups in 127 patients with analyzable culprit OCT images (*p* = 0.390), including 86 with plaque ruptures, 34 with plaque erosions, 2 with severe stenosis and 5 with in-stent restenosis.

**Table 1:** Baseline clinical characteristics.

	Patients with lower aLHR (*n* = 66)	Patients with higher aLHR (*n* = 66)	*p*-value
Age, years	53.5 (47.0–63.3)	57.0 (45.0–63.0)	0.913
Male	56 (84.8)	57 (86.4)	0.804
Current smoking	40 (60.6)	38 (57.6)	0.723
Diabetes mellitus	47 (71.2)	47 (71.2)	1.000
Hypertension	31 (47.0)	37 (56.1)	0.296
Dyslipidemia	37 (56.1)	48 (72.7)	0.046
Previous MI	1 (1.5)	5 (7.6)	0.210
Previous PCI	2 (3.0)	3 (4.5)	1.000
Previous stroke	6 (9.1)	9 (13.6)	0.411
Clinical presentation			1.000
STEMI	58 (87.9)	58 (87.9)	
NSTE-ACS	8 (12.1)	8 (12.1)	
Medications at discharge			
Aspirin	66 (100.0)	65 (98.5)	1.000
Clopidogrel	18 (27.3)	19 (28.8)	0.846
Ticagrelor	48 (72.7)	47 (71.2)	0.846
Statin	65 (98.5)	65 (98.5)	1.000
Atorvastatin 20 mg/day	45 (68.2)	41 (62.1)	0.465
Rosuvastatin 10 mg/day	20 (30.3)	24 (36.4)	0.460
ACEI/ARB	40 (60.6)	40 (60.6)	1.000
Beta-blockers	37 (56.1)	38 (57.6)	0.861

Values are mean ± SD, median (interquartile range) or *n* (%). aLHR, achieved low-density lipoprotein cholesterol to high-density lipoprotein cholesterol ratio; MI, myocardial infarction; PCI, percutaneous coronary intervention; STEMI, ST-segment elevation myocardial infarction; NSTE-ACS, non-ST-segment elevation acute coronary syndrome; ACEI, angiotensin-converting enzyme inhibitor; ARB, angiotensin receptor blocker.

As for the lipid lowering therapy, 98.5% (130 of 132) patients received statins medications at discharge, including 86 cases with atorvastatin 20 mg/day and 44 cases with rosuvastatin 10 mg/day. The detailed category and intensity of statins were similar between the high aLHR group and the low aLHR group. 127 of 132 patients were ongoing statins when re-assessed the lipid profile at 1-year follow-up, resulting a 3.8% of statin discontinuation (4 patients in the high aLHR group and 1 patient in the low aLHR group).

Almost all patients received dual anti-platelet therapy (aspirin plus ticagrelor or clopidogrel) at discharge, the rate of which was comparable between the two groups. Overall, 56.8% of patients were discharged on beta-blockers and 60.6% on angiotensin converting enzyme inhibitor or angiotensin receptor blocker, which were also similar between groups.

### Serum lipid findings at baseline and 1 year

The result of serum lipid levels was shown in Table 2. On admission, there were higher total cholesterol, LDL-C and LHR levels (2.41 [2.16–3.27] mmol/L vs. 2.10 [1.57–2.69] mmol/L, *p* < 0.001) in the high aLHR group. Within 1 year after discharge, the overall proatherogenic lipid levels in both groups improved to a certain extent. Patients with low aLHR showed more reduction in total cholesterol, triglycerides and LHR, when compared with those with high aLHR (LHR reduction: 0.88 ± 0.67 in the low group and 0.47 ± 0.81 in the high group, *p* = 0.002). A higher tendency in the reduction of LDL-C was also observed in the low aLHR group. At 1-year follow-up, all indicators of serum lipids in the high aLHR group were significantly higher than those with low aLHR.

**Table 2:** Baseline and follow-up data of serum lipid levels.

	Patients with lower aLHR (*n* = 66)	Patients with higher aLHR (*n* = 66)	*p*-value
Baseline			
TC, mmol/L	4.52 ± 0.88	4.97 ± 1.28	0.022
TG, mmol/L	1.52 (1.04–2.53)	1.61 (1.17–2.16)	0.927
LDL-C, mmol/L	2.65 (2.17–3.12)	3.05 (2.34–4.07)	0.002
HDL-C, mmol/L	1.30 (1.03–1.51)	1.16 (1.02–1.45)	0.219
LHR	2.10 (1.57–2.69)	2.41 (2.16–3.27)	<0.001
Changes from baseline to 1-year follow-up			
TC, mmol/L	−1.37 (−1.83–0.63)	−0.73 (−1.68–0.30)	0.015
TG, mmol/L	−0.17 (−0.71–0.31)	0.14 (−0.54–0.82)	0.046
LDL-C, mmol/L	−1.08 ± 0.83	−0.76 ± 1.16	0.079
HDL-C, mmol/L	−0.01 ± 0.31	−0.10 ± 0.28	0.103
LHR	−0.88 ± 0.67	−0.47 ± 0.81	0.002
1-year follow-up			
TC, mmol/L	3.08 (2.83–3.54)	4.20 (3.29–4.74)	<0.001
TG, mmol/L	1.30 (1.02–2.08)	1.72 (1.29–2.07)	0.024
LDL-C, mmol/L	1.48 (1.37–1.71)	2.40 (1.81–2.98)	<0.001
HDL-C, mmol/L	1.26 (1.05–1.42)	1.13 (0.94–1.29)	0.005
aLHR	1.28 (1.05–1.42)	2.06 (1.80–2.58)	<0.001

Values are mean ± SD or median (interquartile range) or *n* (%). aLHR, achieved low-density lipoprotein cholesterol to high-density lipoprotein cholesterol ratio; TC, total cholesterol; TG, triglycerides; LDL-C, low-density lipoprotein cholesterol; HDL-C, high-density lipoprotein cholesterol; LHR, low-density lipoprotein cholesterol to high-density lipoprotein cholesterol ratio.

### Characteristics and evolutions of lipid-rich plaques

Baseline angiographic and OCT characteristics of the targeted lipid-rich plaques was listed in Table 3. The average number of plaques were comparable between the two groups (1.64 ± 0.84 vs. 1.62 ± 0.97, *p* = 0.924). Among the 215 lipid-rich plaques, 84 (39.1%) located in the right coronary artery, 83 (38.6%) in the left anterior descending artery and 48 (22.3%) in the left circumflex artery. 38.6% of the non-culprit lipid-rich plaques located in the culprit artery. The distribution of lesion locations was similar between the high and low aLHR groups. With respect to luminal characteristics and lipid components, such as lumen area, area stenosis, lesion length, lipid arc and FCT, there were no statistically significant difference between the two groups. Furthermore, microstructures were also comparable, except a higher prevalence of microchannel (47 [43.9%] vs. 33 [30.6%], *p* = 0.043) and a higher tendency of spotty calcification (55 [51.4%] vs. 42 [38.9%], *p* = 0.065) in the high aLHR group.

**Table 3:** Baseline angiographic and OCT characteristics of the lipid-rich plaques.

	Plaques with lower aLHR (*n* = 108)	Plaques with higher aLHR (*n* = 107)	*p*-value
Lesion location			0.752
LAD	39 (36.1)	44 (41.1)	
LCX	25 (23.1)	23 (21.5)	
RCA	44 (40.7)	40 (37.4)	
Plaques located in the culprit vessel	40 (37.0)	43 (40.2)	0.635
Distance to the ostium, mm	26.0 (14.3–39.6)	24.9 (15.8–40.4)	0.597
Reference lumen area, mm^2^	8.45 (6.89–11.04)	8.72 (6.76–10.54)	0.677
MLA, mm^2^	4.59 (2.99–6.24)	4.27 (2.81–6.16)	0.611
AS, %	49.1 ± 14.8	49.7 ± 16.3	0.798
Mean lumen area, mm^2^	6.73 (4.75–8.28)	6.28 (4.88–8.44)	0.906
Lesion length, mm	13.4 (10.3–19.2)	14.0 (10.4–19.2)	0.819
Mean lipid arc, °	157.6 (128.7–199.5)	155.4 (131.7–190.4)	0.693
Maximum lipid arc, °	210.1 (155.7–287.0)	221.8 (160.1–281.7)	0.887
FCT, µm	90.0 (60.0–130.0)	90.0 (60.0–120.0)	0.638
Lipid length, mm	8.7 (6.1–12.7)	8.1 (5.6–12.4)	0.606
Lipid index	1,357.4 (929.5–2,171.2)	1,350.4 (751.9–2,054.1)	0.544
TCFA	36 (33.3)	41 (38.3)	0.446
Macrophage	88 (81.5)	84 (78.5)	0.585
Microchannel	33 (30.6)	47 (43.9)	0.043
Cholesterol crystal	30 (27.8)	36 (33.6)	0.351
Layered plaque	50 (46.3)	53 (49.5)	0.635
Spotty calcification	42 (38.9)	55 (51.4)	0.065
CLIMA-defined high-risk plaque	9 (8.3)	7 (6.5)	0.617

Values are mean ± SD or median (interquartile range) or *n* (%). OCT, optical coherence tomography; aLHR, achieved low-density lipoprotein cholesterol to high-density lipoprotein cholesterol ratio; LAD, left anterior descending artery; LCX, left circumﬂex artery; RCA, right coronary artery; MLA, minimal lumen area; AS, area stenosis; FCT, fibrous cap thickness; TCFA, thin-cap fibroatheroma.

**Figure 1:**
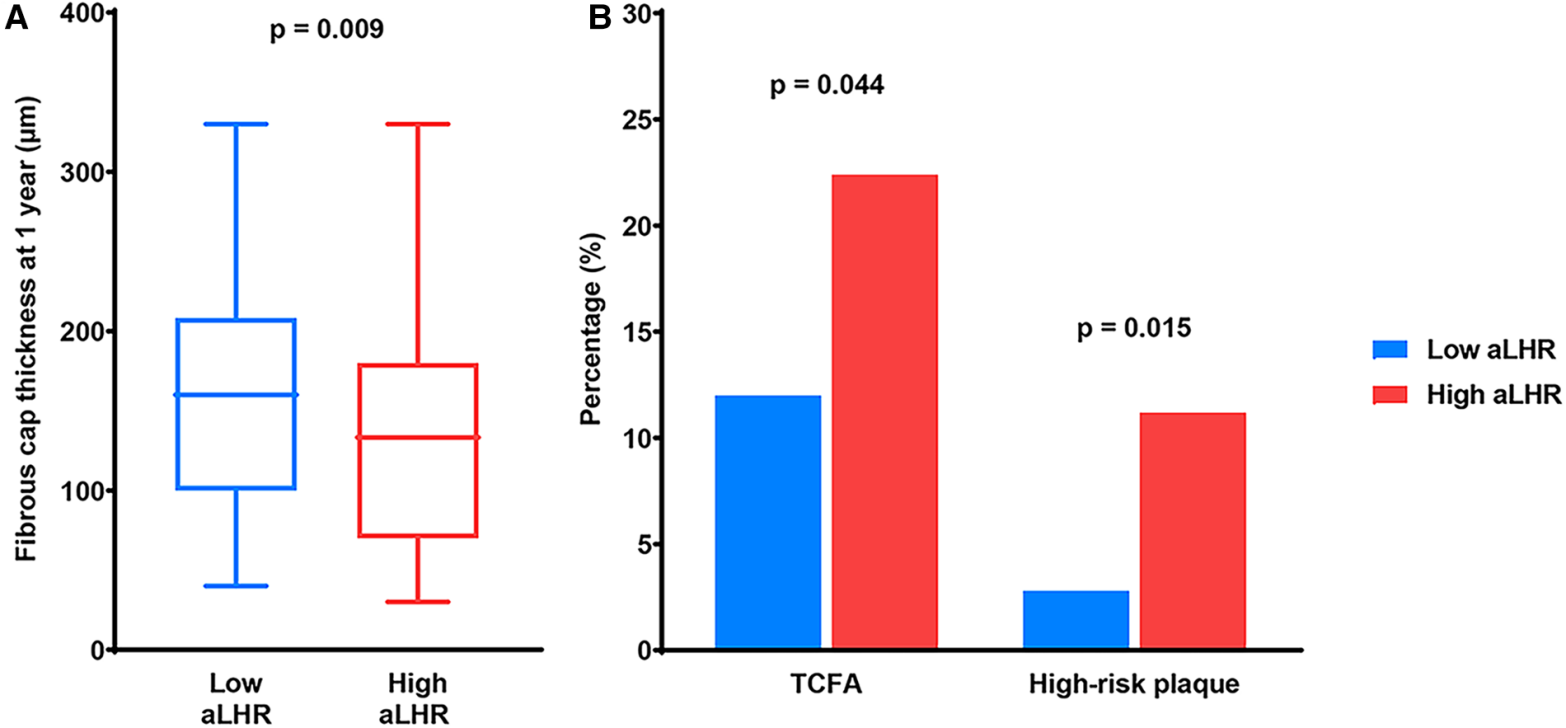
OCT findings at 1-year follow-up. (**A**) Quantitative analysis showed a thinner fibrous cap thickness in patients with high aLHR than those with low aLHR. (**B**) Qualitative analysis showed higher prevalence of TCFA and CLIMA-defined high-risk plaques in the high aLHR group than the low aLHR group. OCT, optical coherence tomography; aLHR, achieved low-density lipoprotein cholesterol to high-density lipoprotein cholesterol ratio; TCFA, thin-cap fibroatheroma.

1-year OCT findings were listed in Figure 1 and Table 4. The FCT was significantly thinner (133.3 [70.0–180.0] µm vs. 160.0 [100.0–208.3] µm, *p* = 0.025) in patients with high aLHR. Besides, there were higher incidences of TCFA (24 [22.4%] vs. 13 [12.0%], *p* = 0.044) and CLIMA-defined high-risk plaques (12 [11.2%] vs. 3 [2.8%], *p* = 0.015) in the high aLHR group. Luminal characteristics, lipid arc and lipid length were comparable between the two groups.

**Table 4:** Follow-up angiographic and OCT characteristics.

	Plaques with lower aLHR (*n* = 108)	Plaques with higher aLHR (*n* = 107)	*p*-value
MLA, mm^2^	4.35 (2.70–5.91)	3.85 (2.34–5.77)	0.266
AS, %	50.1 ± 15.5	51.8 ± 17.6	0.441
Mean lumen area, mm^2^	6.71 (4.62–8.08)	6.07 (4.54–8.11)	0.597
Lesion length, mm	13.3 (10.2–18.7)	13.6 (10.8–18.0)	0.746
Mean lipid arc, °	134.8 (115.9–171.3)	143.4 (116.4–183.6)	0.328
Maximal lipid arc, °	168.2 (139.8–245.0)	186.0 (144.1–267.1)	0.143
Lipid length, mm	6.9 (4.4–10.0)	7.7 (4.6–11.5)	0.240
Lipid index	1,019.6 (595.5–1,547.0)	1,083.5 (653.8–2,051.6)	0.304
Macrophage	82 (75.9)	81 (75.7)	0.969
Microchannel	39 (36.1)	49 (45.8)	0.149
Cholesterol crystal	34 (31.5)	43 (40.2)	0.183
Layered plaque	56 (51.9)	63 (58.9)	0.300
Spotty calcification	46 (42.6)	55 (51.4)	0.196

Values are mean ± SD or median (interquartile range) or *n* (%). OCT, optical coherence tomography; aLHR, achieved low-density lipoprotein cholesterol to high-density lipoprotein cholesterol ratio; MLA, mean lumen area; AS, area stenosis; FCT, fibrous cap thickness; TCFA, thin-cap fibroatheroma.

**Figure 2:**
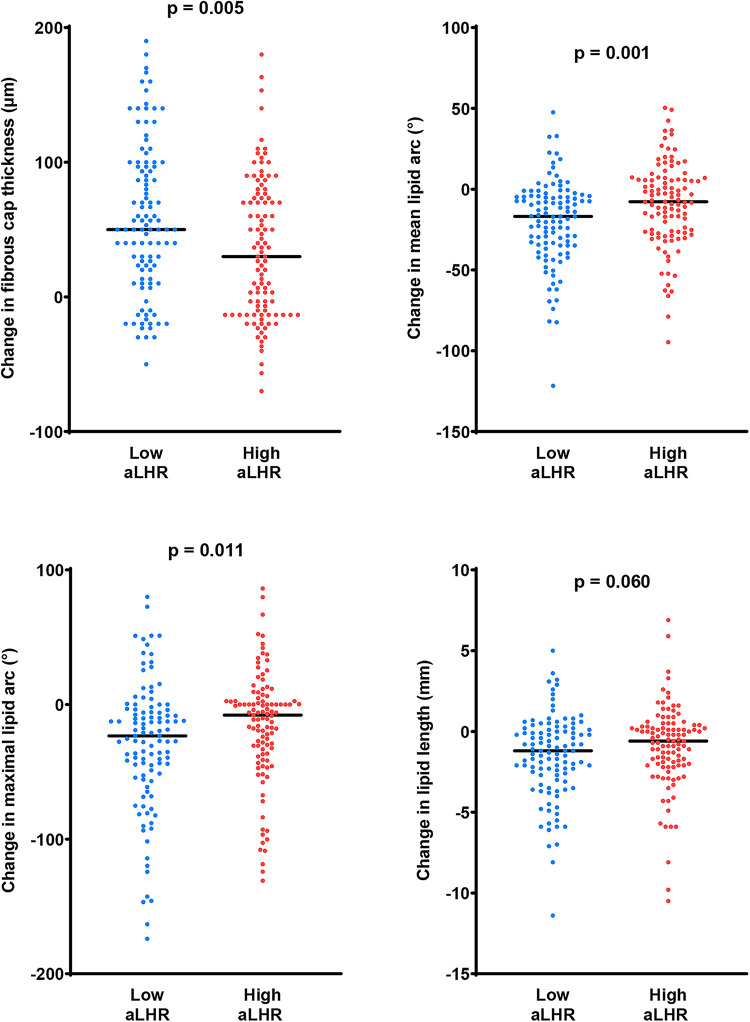
Changes in fibrous cap thickness and lipid components within 1 year. Compared with the high aLHR group, there were more favorable changes in fibrous cap thickness and lipid components in the low aLHR group. aLHR, achieved low-density lipoprotein cholesterol to high-density lipoprotein cholesterol ratio.

The changes in quantitative OCT parameters were illustrated in Figure 2. There were significantly better evolutions of lipid components in the low aLHR group, including a greater reduction in mean lipid arc (16.8 [4.4–34.7] ° vs. 7.8 [−6.0–26.4] °, *p* = 0.001), maximal lipid arc (23.3 [3.8–52.4] ° vs. 7.9[−2.6–36.4] °, *p* = 0.011) and lipid index (306.0 [44.4–609.3] vs. 171.3 [−101.7–489.5], *p* = 0.010). Meanwhile, patients with low aLHR showed apparently better increasement of FCT (50.0 [15.0–96.7] µm vs. 30.0 [−13.3–73.3] µm, *p* = 0.005) within 1 year. There were no significant differences between the two groups in terms of luminal changes.

### Predictive value of aLHR to plaque evolution and vulnerable phenotypes

Changes in vulnerable features of the lipid-rich plaques with baseline and follow-up serum lipid levels were tested for simple linear correlations analysis (Supplementary Table S1) and partial correlations analysis (Table 5). Baseline age, gender, dyslipidemia, hypertension, diabetes mellitus and statins at discharge were taken into partial correlations analysis. Compared with baseline values, serum lipid levels (especially LDL-C and LHR) at 1 year showed better correlation with the changes in OCT-detected lipid components in both models. Among these serum lipid indexes at 1-year follow-up, aLHR had the strongest correlation with the changes in plaque vulnerability in partial correlations analysis: ▵mean lipid arc (*r* = 0.246; *p* < 0.001), ▵maximal lipid arc (*r* = 0.217, *p* = 0.002), ▵FCT (*r* = −0.267, *p* < 0.001), ▵lipid length (*r* = 0.197, *p* = 0.004), ▵lipid index (*r* = 0.193, *p* = 0.005) and 1-year FCT (*r* = −0.282, *p* < 0.001).

**Table 5:** The correlation matrix and coefficient of serum lipid levels with parameters reflecting plaque vulnerability in partial correlation analysis.

	▵ Mean lipid arc	▵ Maximal lipid arc	▵ FCT	▵ Lipid length	▵ Lipid index	1-year FCT
BL TC	−0.042	−0.053	0.068	0.074	−0.008	0.082
BL TG	−0.035	0.000	0.043	−0.129	−0.110	0.022
BL LDL-C	−0.015	−0.019	0.033	0.171*	0.071	0.039
BL HDL-C	−0.008	−0.082	0.088	−0.004	−0.007	0.121
BL LHR	0.013	0.060	−0.059	0.218**	0.115	−0.060
FU TC	0.082	0.025	−0.154*	0.075	0.058	−0.169*
FU TG	−0.008	−0.045	−0.039	−0.030	−0.025	0.013
FU LDL-C	0.205**	0.156*	−0.186**	0.141*	0.129	−0.235**
FU HDL-C	−0.084	−0.128	0.143*	−0.111	−0.120	0.074
aLHR	0.246**	0.217**	−0.267**	0.197**	0.193**	−0.282**

BL, baseline; TC, total cholesterol; TG, triglycerides; LHR, low-density lipoprotein cholesterol to high-density lipoprotein cholesterol ratio; LDL-C, low-density lipoprotein cholesterol; HDL-C, high-density lipoprotein cholesterol; FU, follow-up; aLHR, achieved low-density lipoprotein cholesterol to high-density lipoprotein cholesterol ratio; MLA, mean lumen area; AS, area stenosis; FCT, fibrous cap thickness.

* *p* < 0.05; **p < 0.01.

Using ROCs, the best cut-off value of aLHR to predict the increasement of maximal lipid arc ≥0° was 1.51 [sensitivity: 0.701, specificity: 0.547, Youden index: 0.248, AUC: 0.636, 95% (confidence interval) CI: 0.555 to 0.716, *p* = 0.001]. Coincidentally, the best cut-off value of aLHR to predict the reduction of FCT >0 µm was also 1.51 (sensitivity: 0.709, specificity: 0.531, Youden index: 0.240, AUC: 0.635, 95% CI: 0.547–0.723, *p* = 0.003).

**Figure 3:**
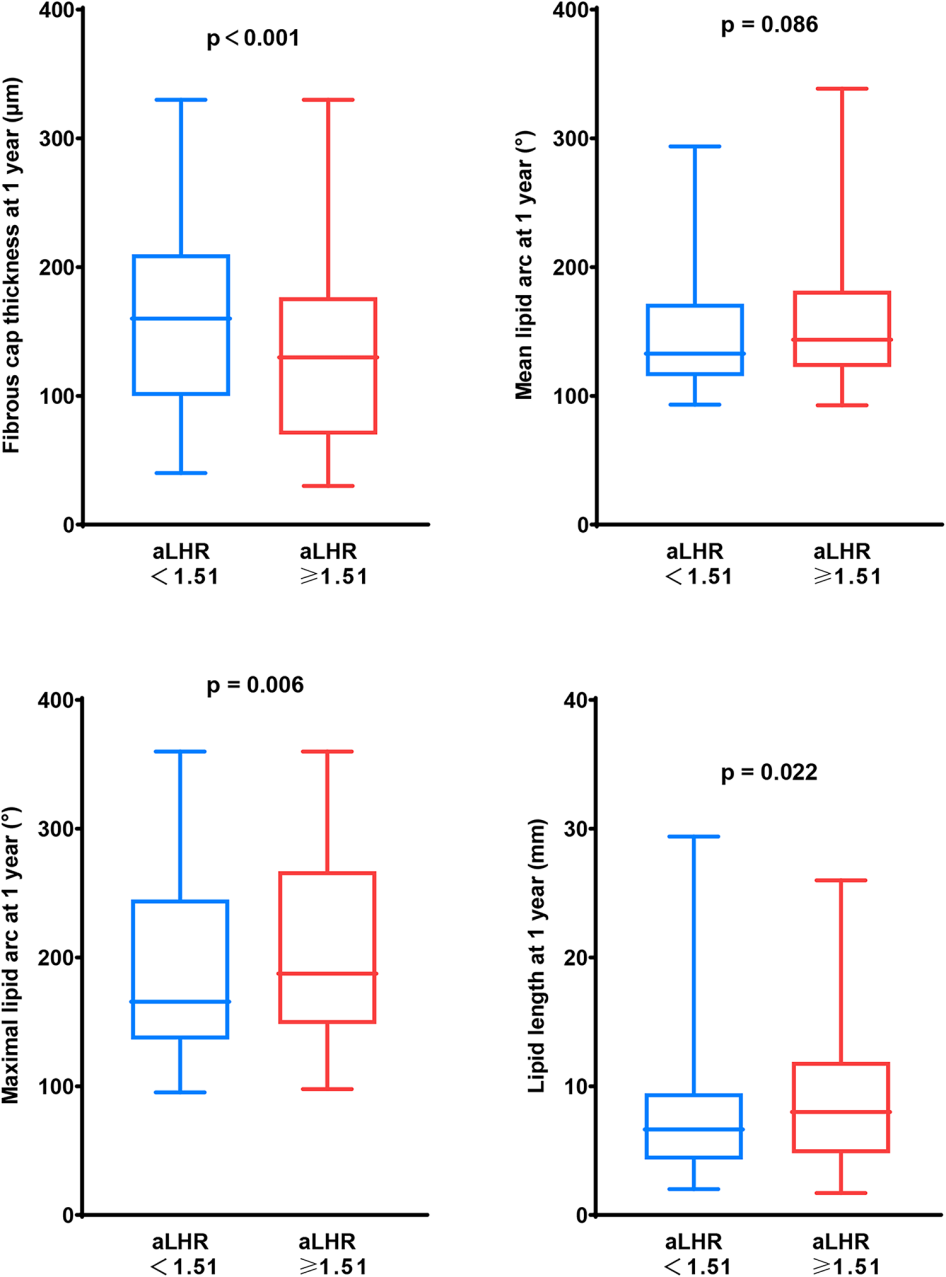
Vulnerability of lipid-rich plaques in patients with aLHR <1.51 and those with aLHR ≥1.51 at 1-year follow-up. At 1-year follow-up, patients with aLHR <1.51 showed thicker a fibrous cap and smaller lipid core than those with aLHR ≥1.51. aLHR, achieved low-density lipoprotein cholesterol to high-density lipoprotein cholesterol ratio.

After adjustment for baseline age, gender, dyslipidemia, hypertension, diabetes mellitus and statins at discharge, 1-year aLHR ≥1.51 was an independent predictor of TCFA [odds ratio (OR): 3.008, 95% CI: 1.370 to 6.605, *p* = 0.006] during 1-year OCT examination. There were larger maximal lipid arc, lipid length and thinner FCT in patients with aLHR ≥1.51 than those with aLHR <1.51 at 1 year (Figure 3).

## Discussion

The relationship between baseline LHR and major adverse cardiovascular events has been investigated in ACS patients after percutaneous coronary intervention (20). However, evidence on relationship between aLHR and the evolution of untreated non-culprit plaques was poor. This study revealed for the first time the predictive value of aLHR for the evolution of lipid-rich plaques in patients with ACS. Patients with higher aLHR showed more aggressive progression on lipid components and FCT, resulting higher prevalence of vulnerable plaque phenotypes at 1-year follow-up. Compared with single serum lipidic index at baseline or follow-up, aLHR showed the best correlation with the progression of lipid-rich plaques. aLHR ≥1.51 was an independent predictor of TCFA at 1 year.

### Importance and potential mechanisms of aLHR in ACS

Previous studies mainly focused on the impact of baseline serum lipid levels on clinical prognosis, which is undoubtedly a very important aspect (20, 21). However, for ACS patients, they would be asked to start taking antiplatelet and lipid-lowing medications on a long-term and regular basis after discharge. These secondary preventive medications would cause significant changes in index serum lipoproteins, making them difficult to reflect the serum lipid levels during follow-up accurately, just as shown in Table 2 of current study. Meanwhile, several recent studies have shown that greater visit-to-visit lipid variability and better achieved atherogenic lipoproteins significantly associate with coronary atheroma progression and clinical outcomes, which was consistent with the main findings of our study and highlights the benefits of achieving low and consistent atherogenic lipoprotein levels during follow-up (2–4).

Accounting for approximately two thirds of plasma cholesterol, LDL-C has been recognized as the primary driver of atherogenesis (22). HDL-C was recognized as a potential modulator of LDL-C atherogenicity and might attenuate the atherogenic drive of LDL-C particles in plaque progression (22). As a derived ratio of LDL-C and HDL-C, LHR could reflect the balance level between the cholesterol carried by atherogenic and protective lipoproteins concisely. Patients with the same total cholesterol value may show significantly different LHR levels and there might be significantly higher cardiovascular risks for those with higher LHR levels, owing to the imbalance between the detailed lipoproteins. As shown in the Person correlation analysis and partial correlations analysis of current study, aLHR had better correlation with the evolution of lipid components, when compared with either of them.

In addition to LDL-C, HDL-C and the derived LHR, progression or regression of coronary atheroma was also influenced by other lipoproteins. The EVAPORATE trial proved that eicosapentaenoic acid could impact plaque changes in those with elevated triglycerides on statin therapy, indicating effect of triglyceride on plaque composition (23). In current study, baseline triglyceride was also correlated with changes of lipid length and lipid index in Person correlation analysis. Meanwhile, this correlation was not statistically significant in follow-up triglyceride levels and in partial correlations analysis. Underlying mechanisms of each lipoprotein on plaque changes and the interactions of them need to be further explored in future studies.

### Clinical significance of OCT-detected vulnerable plaques

The size of necrotic core and thickness of fibrous cap were critical indicators of plaque vulnerability. A combined OCT and intravascular ultrasound study demonstrated that fibrous cap thickness <52 mm is a critical morphological discriminator for plaque rupture (24). From a 4-year follow-up of 1,474 patients with OCT examination of the target vessel, researchers found that the presence of lipid-rich plaques increased risk for adverse clinical outcomes, especially in those with longer lipid length, larger lipid arc, and higher area stenosis (12). Kubo et al. also pointed out that non-culprit plaques with a larger maximum lipid arc, thinner FCT and smaller minimum lumen area were independent predictors of subsequent ACS in the median follow-up period of 6 years (14). Meanwhile, the CLIMIA study demonstrated the simultaneous presence of MLA <3.5 mm^2^, FCT <75 µm, maximum lipid arc >180° and macrophages were strongly associated with increased risk of major cardiovascular events at 1-year follow-up (17). In addition, a recent study published in American Journal of Preventive Cardiology pointed out that in the advanced subclinical atherosclerosis stage, more than 50% of patients suffer an ischemic event without obvious stenosis but a high plaque burden (25). As a luminal examination, coronary angiography could not detect plaque burden and lipid components, making it unbale to predict this high-risk subclinical status accurately. All of these findings highlight the crucial significance of detecting and managing patients with vulnerable plaque phenotypes, especially the maximum lipid arc and FCT in OCT.

### Implications of aLHR in predicting evolution of lipid-rich plaques

A significant association between aLHR and progression of lipid arc and FCT was shown in the linear regression analysis. Even compared with the classic lipid indexes (such as LDL-C and triglycerides), we found aLHR was the strongest predictor of plaque progression and vulnerable plaque phenotype at 1 year. Kurabayashi et al. suggested the target index for regression of atherosclerosis should be less than 2.0 for primary prevention and less than 1.5 for secondary prevention (26). Based on the ROC curves, the cut-off point was 1.51 for secondary prevention of ACS in our study, very close to their previous studies. Due to the well-known impact of age, gender, hypertension, dyslipidemia and diabetes mellitus on the progression of atherosclerotic plaques, we adjusted for them and proved the independent predictive value of aLHR on the presence of TCFA at follow-up.

As one of the most important secondary cardiovascular prevention targets, adequate lipid control was still not achieved in majority of ACS, highlights the urgent necessity of whole-process lipid management (27). Our data revealed the clinical significance and underlying mechanisms of achieving LHR during follow-up. In daily practice, monitoring serum lipoproteins regularly and maintaining aLHR less than 1.51 might predict a favorable regression of plaque vulnerability. For those with aLHR over 1.51, we should adjust lipid-lowering drugs in time, conduct more stringent control of aLHR, and conduct further non-invasive or invasive examinations and interventions if necessary.

Notably, the protective effect of HDL-C in its extremely high levels is controversial. A large-scale Japanese cohort study revealed that approximately 1.5% of individuals exhibit an extremely high HDL-C level (≥2.33 mmol/L), and they were associated with adverse outcomes (28). Coincidentally, all patients showed HDL-C level less than 2.33 mmol/L in current study, which might be related to selection basis and racial differences. Therefore, current findings on the relationship between aLHR and plaque evolution might not be suitable for those with extremely high HDL-C levels.

### Study limitations

First of all, as a retrospective single-center study, potential bias in patient and lesion selection was inevitable. Only patients survived and finished OCT scan at follow-up were enrolled in this study. We adjusted for age, gender, cardiovascular risk factors and statins at discharge to diminish the possible influences of confounders in the generalized estimating equations. In addition, the intervention of lifestyles also influent the evolution of targeted plaques, and some of these data was not available in this study for retrospective reasons. Besides, only patients with non-culprit lipid-rich plaques were enrolled into analysis. The effects of aLHR on fibrous and calcified plaques needs further exploration. Fourth, the lipid lowering therapy of most enrolled patients were moderate-intensity statins, and none of them received high-intensity statins or PCSK9 inhibitors in current study. This may attribute to the genetic differences of Chinese population in genetics and weight, and the clinical practice at discharge. Further studies were warranted to explore the application of aLHR in patients with different dose of statins or PCSK9 inhibitors. Fifth, limited by the relatively small sample size, the impact of kidney function on the change of plaque components (especially calcification) was not analyzed in detail in current study.

## Conclusions

In this serial OCT study focusing on the impact of aLHR on the evolution of lipid-rich plaques in ACS, patients with higher aLHR showed more aggressive changes in lipid components and more vulnerable plaque phenotype at 1-year follow-up. These findings highlight the importance of standardized aLHR control and provided evidence for individualized secondary prevention of ACS.

## Data Availability

The raw data supporting the conclusions of this article will be made available by the authors, without undue reservation.
