# Supplementary material for: Achieved low-density lipoprotein cholesterol to high-density lipoprotein cholesterol ratio predicts the pathophysiological evolution of lipid-rich plaques in acute coronary syndromes: an optical coherence tomography study
Source: Front Cardiovasc Med. 2023 Jul 10;10:1181074. doi: 10.3389/fcvm.2023.1181074 (PMC10369790; doi:10.3389/fcvm.2023.1181074)
Supplement: Supplementary file 1 [file Table1.docx]

**Supplementary Table 1. The correlation matrix and coefficient of serum lipid levels with parameters reflecting plaque vulnerability in simple linear analysis.**

|  | △ Mean lipid arc | △Maximal lipid arc | △ FCT | △ Lipid length | △ Lipid index | 1-year FCT |
| --- | --- | --- | --- | --- | --- | --- |
| BL TC | -0.062 | -0.075 | 0.117 | 0.009 | -0.041 | 0.143* |
| BL TG | -0.071 | -0.047 | 0.051 | -0.170* | -0.144* | 0.022 |
| BL LDL-C | -0.028 | -0.035 | 0.081 | 0.115 | 0.045 | 0.108 |
| BL HDL-C | -0.018 | -0.064 | 0.115 | 0.010 | 0.007 | 0.161* |
| BL LHR | 0.008 | 0.033 | -0.021 | 0.141* | 0.075 | -0.009 |
| FU TC | 0.091 | 0.028 | -0.123 | 0.065 | 0.053 | -0.132 |
| FU TG | -0.019 | -0.061 | -0.051 | -0.063 | -0.050 | -0.015 |
| FU LDL-C | 0.210** | 0.149* | -0.141* | 0.113 | 0.115 | -0.189** |
| FU HDL-C | -0.085 | -0.107 | 0.147* | -0.066 | -0.092 | 0.097 |
| aLHR | 0.246** | 0.199** | -0.224** | 0.146* | 0.163* | -0.241** |

*P < 0.05; **P < 0.01. BL, baseline; TC, total cholesterol; TG, triglycerides; LHR, low-density lipoprotein cholesterol to high-density lipoprotein cholesterol ratio; LDL-C, low-density lipoprotein cholesterol; HDL-C, high-density lipoprotein cholesterol; FU, follow-up; aLHR, achieved low-density lipoprotein cholesterol to high-density lipoprotein cholesterol ratio; MLA, mean lumen area; AS, area stenosis; FCT, fibrous cap thickness.
